# Novel *In Situ* Pretreatment Method for Significantly Enhancing the Signal In MALDI-TOF MS of Formalin-Fixed Paraffin-Embedded Tissue Sections

**DOI:** 10.1371/journal.pone.0041607

**Published:** 2012-08-10

**Authors:** Yu Kakimoto, Tatsuaki Tsuruyama, Takushi Yamamoto, Masaru Furuta, Hirokazu Kotani, Munetaka Ozeki, Akihiko Yoshizawa, Hironori Haga, Keiji Tamaki

**Affiliations:** 1 Department of Forensic Medicine and Molecular Pathology, Graduate School of Medicine, Kyoto University, Kyoto, Japan; 2 Analytical and Measuring Instruments Division, Kyoto Applications Development Center, Shimadzu Corporation, Kyoto, Japan; 3 Department of Diagnostic Pathology, Graduate School of Medicine, Shinshu University Hospital, Matsumoto, Japan; 4 Department of Diagnostic Pathology, Graduate School of Medicine, Kyoto University Hospital, Kyoto, Japan; Moffitt Cancer Center, United States of America

## Abstract

The application of matrix-assisted laser desorption/ionization (MALDI)-based mass spectrometry (MS) to the proteomic analysis of formalin-fixed paraffin-embedded (FFPE) tissue presents significant technical challenges. *In situ* enzymatic digestion is frequently used to unlock formalin-fixed tissues for analysis, but the results are often unsatisfactory. Here, we report a new, simplified *in situ* pretreatment method for preparing tissue sections for MS that involves heating with vapor containing acetonitrile in a small airtight pressurized space. The utility of the novel method is shown using FFPE tissue of human colon carcinoma. The number and intensity of MALDI peaks obtained from analysis of pretreated tissue was significantly higher than control tissue not subjected to pretreatment. A prominent peak (*m/z* 850) apparently specific to cancerous tissue was identified as a fragment of histone H2A in FFPE tissue pretreated using our method. This highly sensitive treatment may enable MALDI-MS analysis of archived pathological FFPE samples, thus leading to the identification of new biomarkers.

## Introduction

Human tissues collected during biopsy, surgery, or autopsy are usually preserved as formalin-fixed paraffin-embedded (FFPE) samples. Formaldehyde crosslinks proteins by methylene-bridging of residue side-chains, thereby stabilizing the cellular morphology and preventing autolysis and decomposition [1], [2]. This is problematic for MS studies as the bridging makes it difficult to extract biomolecules and analyze protein structure. Formalin-fixed tissues must therefore be subjected to an “unlocking” process, similar to microwave heating used in immunohistochemistry [3], [4] or reduction of proteins with β-mercaptoethanol used prior to electrophoresis [5], [6]. In MS analysis, enzymatic digestion is classically used to fragment proteins into detectable peptides [7], [8]. However, enzymatic digestion is usually insufficient for demethylation of fixed samples, and results in substantially weaker MS signal intensities and lower signal/noise (S/N) ratios with FFPE tissues compared to freshly frozen tissues [9].

There are a number of reports discussing new procedures for preparing FFPE sections for MALDI-MS. Some researchers performed microdissection of pathologically diagnosed disease regions, followed by protein extraction and digestion, but this technique is costly and time-consuming [9], [10]. Some common surfactants have been shown to improve MS sensitivity, but there is considerable disagreement regarding the chemical species that work best and the proper amounts to use [11], [12], [13]. Experiments involving heating sample slides in a solution of EDTA or citric acid resulted in a several-fold increase in signal intensity with little change in the S/N ratio [14], [15]. However, no consensus has been achieved with respect to the best ways to handle FFPE sections for MALDI-MS.

In the present study, we developed a simple *in situ* pretreatment technique for preparing FFPE sections for MALDI-MS that is rapid and effective, and requires no expensive equipment. Our method involves pretreating tissues at high pressure and temperature for a short period, which is sufficient to enhance permeability but does not cause tissue damage. Applying this method to FFPE colon carcinoma tissue resulted in a significant increase in MS signal intensity and number of ions detected. From these data we identified a specific protein that is highly expressed in cancerous tissue. Our method thus enhances the sensitivity of MALDI-MS in the analysis of FFPE specimens.

## Materials and Methods

### Patients

The study was approved by the Committee of Medical Ethics of the Graduate School of Medicine, Kyoto University. We obtained written informed consent from all participants involved in our study. Six patients were assessed by routine endoscopic biopsy for pathologic diagnosis of colon tumor or surgical excision of colon carcinoma. All patients were 60 years of age; three were men and three were women. Each patient received a surgical colectomy. The pathological diagnosis was well-differentiated tubular adenocarcinoma. Tissue samples were fixed with buffered formaldehyde, dehydrated with ethyl alcohol, and embedded in paraffin. Tissues were sectioned at 4-µm thickness as recommended previously [16], and then mounted on Indium tin oxide-coated (ITO) glass slides at 8–12 ohms (Sigma-Aldrich, St. Louis, MO, USA). The plates were placed in a desiccator at 55°C and allowed to dry overnight.

### Rinsing procedure

Many of the solvents used in histology to dehydrate, fix, and preserve tissues dissolve lipids, and this in turn affects the efficiency of protein desorption/ionization. Washing off lipids from the tissue section is thus important in order to obtain high intensity signals [17], [18]. We performed a four-step deparaffinization procedure: 3 washes in xylene for 5 min each, 2 washes in 100% ethanol for 3 min each, 1 wash in 90% ethanol for 3 min, and 1 wash in 80% ethanol for 3 min. All steps were carried out with stirring at room temperature.

### Swelling incubation and steaming procedure (SSP)

**Figure 1:**
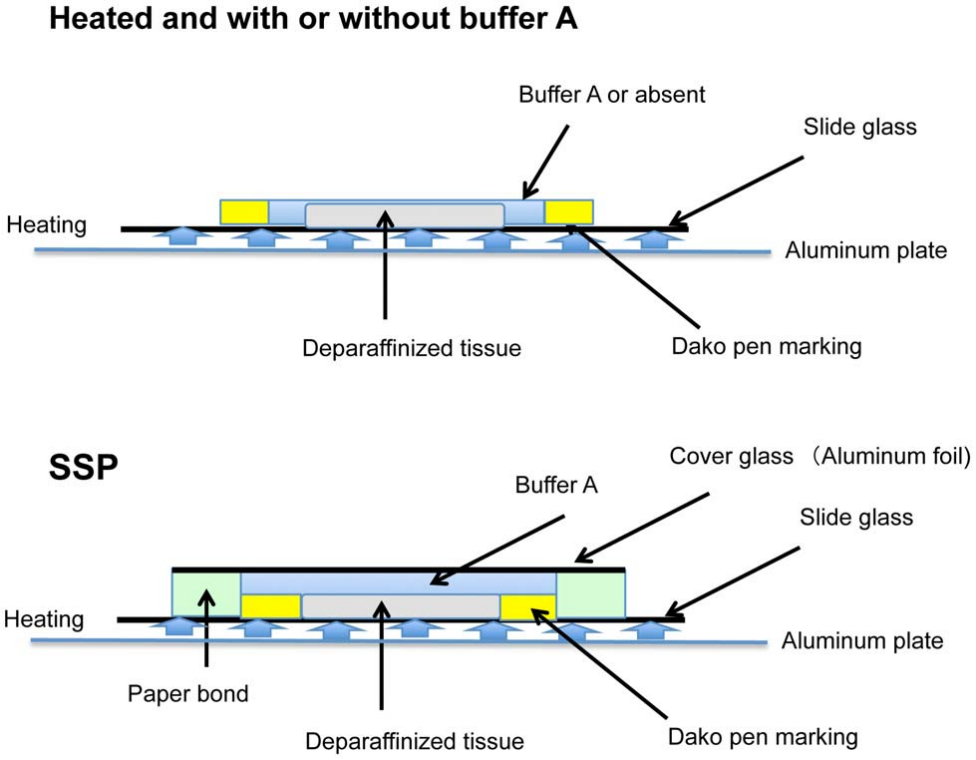
Illustration of pretreatment methods. Upper: open air heating pretreatment with or without Buffer A. Lower: SSP pretreatment.

Sample glass slides were incubated *in situ* for 1 h at 37°C in Buffer A (0.1 M NH_4_HCO_3_ and 30% (v/v) CH_3_CN). We named this step the “swelling incubation”. After removing the buffer, the tissue section on the slide was encircled with paper bond (Ta-100; Kokuyo, Osaka, Japan). The bond became solid in a few minutes and made a small chamber around the sample. Next, the chamber was filled with a volume of Buffer A sufficient to cover the entire surface of the sample. Each slide was then covered with a glass coverslip or aluminum foil to keep it airtight and pressurized, thereby protecting the sample from excessive evaporation. Finally, in the “steaming incubation” step, the slides were heated at 94°C on an aluminum hot plate (DAKO, Glostrup, Denmark) (Figure 1).

### Enzymatic digestion

For digestion, 200 µL of 0.05 µg/µL trypsin (Sequencing Grade Modified Trypsin; Promega, WI, USA) solution containing 2.5 mM NH_4_HCO_3_ and 10% (v/v) CH_3_CN was placed on each slide and the tissue was covered with a glass coverslip. The slides were then incubated *in situ* at 37°C for 24 h, after which the trypsin solution was stripped off and the slides were dried at room temperature.

### Matrix deposition

The matrix solution was prepared by dissolving 2,5-dihydroxybenzoic acid (DHB) in a solution of 50% methanol and 0.1% trifluoroacetic acid. Each sample slide was placed in a slot on a MALDI target plate, affixed with conductive tape, and inserted into a chemical inkjet printer (CHIP-1000; Shimadzu, Kyoto, Japan). Matrix was deposited in 15-nL droplets by micro-spotting 50 cycles of 300 pL on each spot at a spatial interval of 250 µm. After spotting, the target plates were dried at room temperature. More than 12 spots of matrix were deposited on both the normal and cancerous areas of the tissue.

### Mass spectrometry

Mass spectra were acquired using a highly flexible research grade MALDI TOF/TOF mass spectrometer (AXIMA Performance; Shimadzu, Kyoto, Japan) equipped with a 337-nm pulsed nitrogen laser that was operated at a repetition rate of 10 Hz. Spectra were acquired in positive ion mode over the range *m/z* 500–3000. External calibration was performed using a solution of adrenocorticotropic hormone (ACTH) fragment 18–39.

### Tandem mass spectrometry

Tandem mass spectra (MS/MS) were collected using a MALDI-QIT-TOF MS (AXIMA Resonance; Shimadzu, Kyoto, Japan). Spectra were exported to the Mascot search engine (Matrix Science, Boston, MA, USA) using the following search parameters: taxonomy = *Homo sapiens*; database = SwissProt MSDB 20060831; MS tolerance = 0.2 Da; MS/MS tolerance = 0.3 Da; enzyme = trypsin/P; variable modifications = oxidation (M); missed cleavages = 1. Matches were assigned with a significance threshold of *P*≤0.05. In visualization, the raw MALDI-MS data were converted into BioMap format.

### Statistical analysis

Data were analyzed with an unpaired *t*-test using SPSS software (SPSS, Chicago, IL, USA).

## Results

### Swelling and steaming pretreatment enhances MS signal intensity and S/N ratio

**Figure 2:**
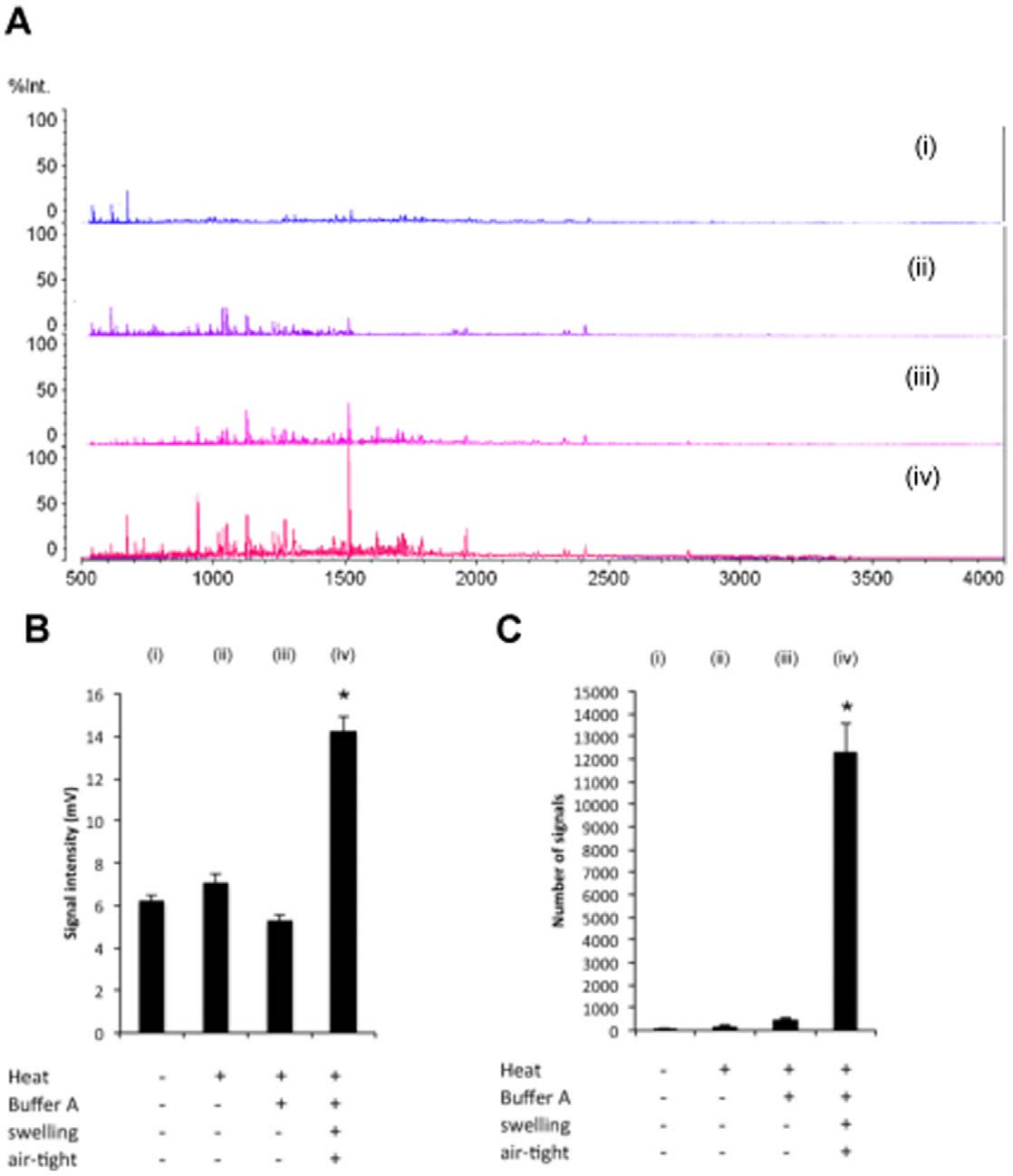
Effect of SSP pretreatment on MS signal intensity and signal-noise ratio. (A) A representative MS profile obtained from analysis of normal areas of a patient's colon tissue. (i) Control tissue, not pretreated; (ii) Tissue heated without Buffer A in the open air; (iii) Tissue heated with Buffer A in the open air; (iv) SSP pretreated tissue, swollen and heated with Buffer A in an airtight space. Heating time was one minute. %Int: the maximum value (100% intensity) corresponds to 14.0 mV. All axes display the same scale. (B) and (C) Effect of heating process on average intensity and number of signals with a signal noise (S/N) ratio>5 across the *m/z* range 500–4000 in analyses of tissue sections (i)–(iv) described above (mean ± SD, n = 6). (iv) corresponds to SSP pretreatment. Signal was significantly enhanced in tissue subjected to SSP pretreatment (*P<0.001). Heating time was one minute (B) and three minutes (C).

Sections of FFPE human colon tissue were pretreated *in situ* as shown in Figure 1. We first compared tissues subjected to the SSP pretreatment with tissues only heated with and without Buffer A in the absence of an airtight seal. Airtight spaces were maintained with cover glass as described in the Materials and Methods. The intensity of MS signals over the *m/z* range 500–4000 was enhanced in colon tissue sections subjected to the SSP pretreatment relative to tissues that were heated in the presence and absence of Buffer A without being sealed with a cover slip (Figure 1; Figures 2A–B). In addition, the S/N ratio was significantly higher in tissue sections subjected to the SSP pretreatment (Figure 2C). These results thus indicate that SSP pretreatment significantly increases both the signal intensity and S/N ratio in MALDI-MS analyses of FFPE tissues.

### The optimal duration of pretreatment heating

**Figure 3:**
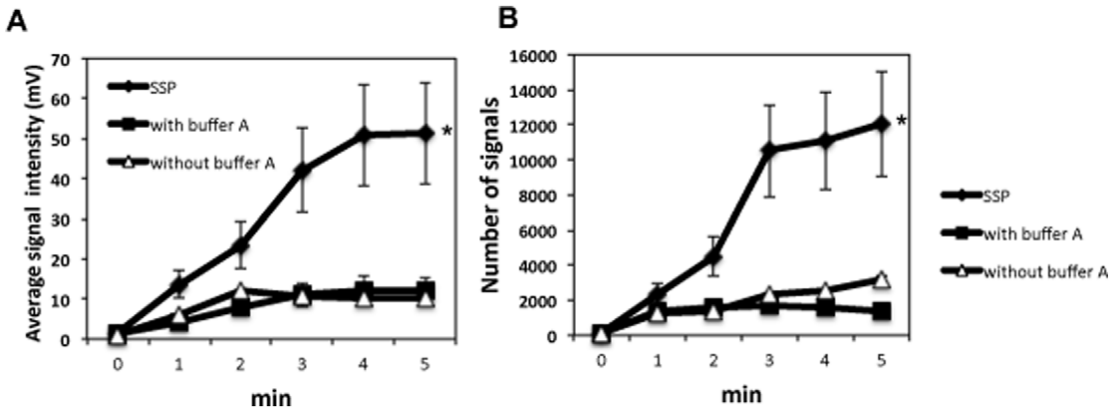
Optimization of SSP pretreatment heating time. Effect of heating duration on average MS signal intensity across the *m/z* range 500–4000 (mean ± SD, n = 6) for normal tissue sections subjected to swelling prior to heating to 94°C for various periods in the presence of Buffer A (SSP) (see Figure 1, lower), and tissue sections not subjected to prior swelling before being heated to 94°C for various periods in the presence or absence of Buffer A (Heated) (see Figure 1, upper). (A) Average signal intensity (mean ± SD, n = 6). (B) Number of signals with a signal-noise ratio (S/N)>5 (mean ± SD, n = 6). Both average signal intensity and S/N ratio were significantly enhanced in tissues subjected to SSP pretreatment at all heating durations (*P<0.001).

To determine the optimal duration of pretreatment heating, tissue sections subjected to SSP pretreatment and unswelled tissues were heated in the open air at 94°C for various time periods and then subjected to MALDI-TOF MS analysis. In SSP pretreated tissues, the MS signal intensity and number of signals with a S/N ratio>5 increased significantly with increasing duration of pretreatment heating; however, the intensity nearly plateaued at a pretreatment heating time of 3 minutes (Figures 3A–B). Beyond 3 minutes of *in situ* heating, the tissue began to peel off the slide glass and the tissue architecture began to disintegrate; therefore, 3 min was selected as the optimal heating time for SSP pretreatment.

### SSP pretreatment resulted in identification of a cancer-specific MS signal

**Figure 4:**
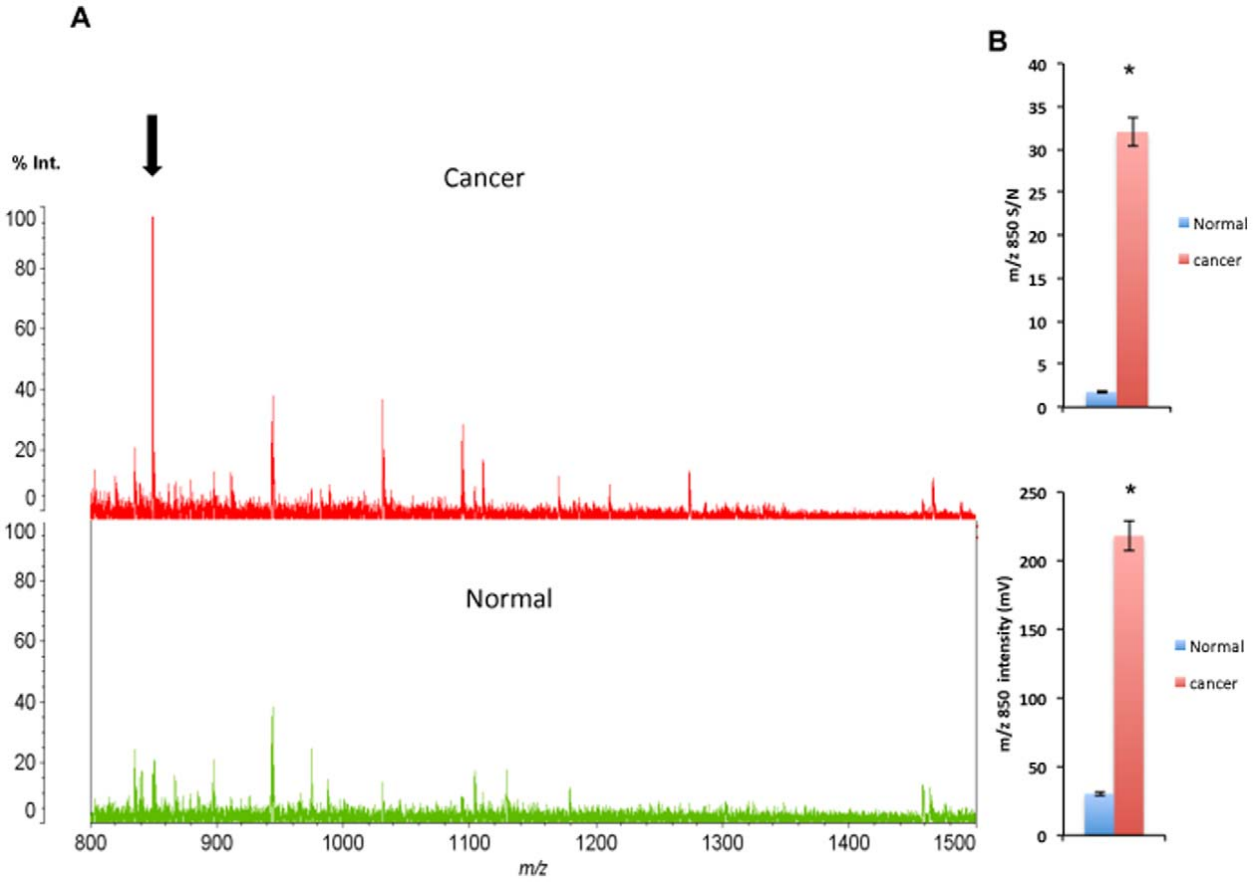
MS profiling of human colon tissue. (A) Representative MS profiles obtained from cancerous and normal areas of a patient's colon tissue. The ion at *m/z* 850 (arrow) is prominent in the cancerous area (red spectrum), but is not obvious in the control (green spectrum). (B) The upper graph shows the S/N ratio of the *m/z* 850 signal obtained from normal and cancerous areas of colon tissue (*P<0.001, cancer vs. normal, n = 6). The lower graph shows the average signal intensity at *m/z* 850 in the normal and cancerous areas of colon tissue (*P<0.001, cancer vs. normal, n = 6).

Pretreated normal colon and adenocarcinoma tissues were analyzed separately by MS across the *m/z* range 500–4000. Representative data for adenocarcinoma and normal mucosa tissue are shown in Figure 4A. The S/N ratio for the *m/z* 850 signal and average intensity of the *m/z* 850 signal were significantly higher in the cancerous areas than in the normal areas (Figure 4B).

**Figure 5:**
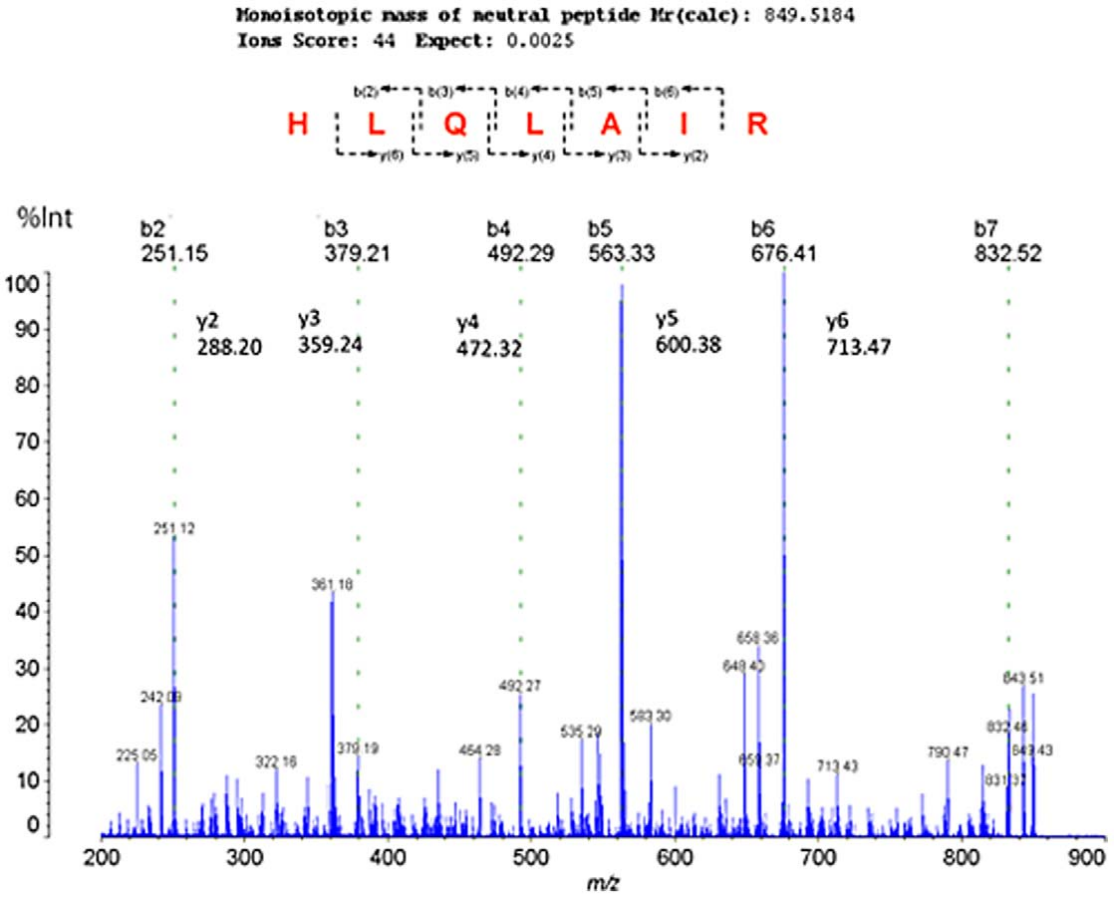
MS profiling of human colon tissue. Tandem mass spectrum of the precursor ion at *m/z* 850. Product ions, b(2∼6) and y(2∼6), were observed, and were determined to represent the sequence HLQLAIR. A database search identified the ion at *m/z* 850 as a fragment of histone H2A (Ion Score = 44, *P* = 0.0025).

The structure of the *m/z* 850 ion was analyzed using MS/MS (Figure 5). Product ions at *m/z* 251, 288, 359, 379, 472, 492, 563, 600, 676, and 713 were produced upon fragmentation of the *m/z* 850 precursor ion, and corresponded to b- or y-series ions derived from the sequence HLQLAIR. A search of the SwissProt database indicated that the precursor ion at *m/z* 850 is a fragment of histone H2A (Ion Score = 44, *P* = 0.0025).

## Discussion

In the present study, we demonstrated that our novel method significantly enhances both the signal intensity and S/N ratio in MS analyses of FFPE tissues. Formalin fixation renders tissues hydrophobic, and therefore it is difficult for aqueous reagents to penetrate the rough and firm surface of FFPE tissues. To improve tissue permeability, we first incubated FFPE specimens in Buffer A for 1 h at 37°C after rinsing. This treatment results in reswelling of the deparaffinized sample and increases the permeability of the tissue.

Next, a steaming procedure under airtight conditions is performed. Once the buffer A penetrates into the hydrophobic tissues, the swollen vaporized buffer component enlarges the space between collagen and other connective tissues, which is expected to enhance subsequent permeability of the tissue section. Such physically enforced enlargement of the tissue may contribute to disruption of the methylene bridges resulting from formalin fixation.

Following the steaming procedure, tissues were heated to retrieve antigens. The use of heating to retrieve antigens in FFPE samples prior to immunohistochemistry has already been established [3], [19]. We utilized a temperature high enough to unlock the formaldehyde crosslinks but of sufficiently short duration so that the specimen's internal molecular composition would remain intact. As shown in Figure 2A, there were no significant differences between control tissues and tissues subjected to the different pretreatments with respect to the overall MS peak profile or the predominant peaks. However, because the similarity of pretreated and control tissue spectra does not necessarily indicate that the internal molecular composition of the pretreated tissues had been maintained, we must evaluate the protein composition of the tissues in a subsequent study. On the other hand, pretreatment enhanced the MS signal intensity, which led to the detection of a greater number of ions above a S/N threshold of 5 as compared to the control tissue.

Trypsin was used in the present study to digest proteins retrieved using our novel procedure. Trypsin cleaves C-terminal to arginine and lysine residues that have the ability to form methylene crosslinks. The most frequent type of crosslink formed by formaldehyde in collagen is between the nitrogen atom at the end of the side-chain of lysine and the nitrogen atom of a peptide linkage, and the number of such crosslinks formed increases over time [20]. Therefore, trypsin is one of the most effective reagents for enhancing the solubility of crosslinked proteins for MS analysis. Our swelling pretreatment procedure enhances the permeability of tissues for tryptic digestion.

Using our methodology, histone H2A was identified as a potential marker of cancerous lesions. The overexpression of histone H2A in colon cancer cells has been reported in studies using LC/MS [21] and RT-PCR based on genomic data [22]. In the present study, sections of normal and cancerous tissues were digested separately in order to avoid cross-contamination of samples, and we confirmed that the signal derived from histone H2A is more intense in FFPE carcinoma tissue, indicating elevated expression. Although we focused only upon the most striking ion to show the superiority of our swelling and steaming pretreatment technique to previous heating-only pretreatment methods, the results still indicate that MS can be a powerful tool for *in situ* screening for metabolic biomarkers. The microdistribution of ionizable components can be determined directly on a tissue section prepared using our pretreatment method. Target molecules can then be identified using MS/MS, thereby providing more specific information about the pathogenesis of a given disease than would otherwise be possible.

In summary, we developed a novel method for pretreating FFPE tissues for MALDI-MS analysis. Pretreating FFPE tissues using our method significantly increases the number of ions observed, the intensity of the ions, and the S/N ratio. Considering the vast numbers of FFPE specimens that are stored in hospitals and laboratories worldwide, our new practical pretreatment method may significantly expand the number of samples amenable to MS-based proteomic analysis.
